# An optical and electrochemical sensor based on l-arginine functionalized reduced graphene oxide

**DOI:** 10.1038/s41598-022-23949-5

**Published:** 2022-11-12

**Authors:** Sanaz Ghanbari, Fatemeh Ahour, Sajjad Keshipour

**Affiliations:** 1 Department of Nanotechnology, Faculty of Chemistry, Urmia University, Urmia, Iran; 2 Institute of Nanotechnology, Urmia University, Urmia, Iran

**Keywords:** Analytical chemistry, Electrochemistry

## Abstract

The electrochemical and photochemical properties of graphene derivatives could be significantly improved by modifications in the chemical structure. Herein, reduced graphene oxide (RGO) was functionalized with l-arginine (l-Arg) by an amidation reaction between the support and amino acid. Deposition of a powerful ligand, l-Arg, on the optically active support generated an effective optical chemosensor for the determination of Cd(II), Co(II), Pb(II), and Cu(II). In addition, l-Arg-RGO was used as an electrode modifier to fabricate l-Arg-RGO modified glassy-carbon electrode (l-Arg-RGO/GCE) to be employed in the selective detection of Pb(II) ions by differential pulse anodic stripping voltammetry (DP-ASV). l-Arg-RGO/GCE afforded better results than the bare GCE, RGO/GCE, and l-Arg functionalized graphene quantum dot modified GCE. The nanostructure of RGO, modification by l-Arg, and homogeneous immobilization of resultant nanoparticles at the electrode surface are the reasons for outstanding results. The proposed electrochemical sensor has a wide linear range with a limit of detection equal to 0.06 nM, leading to the easy detection of Pb(II) in the presence of other cations. This research highlighted that RGO as a promising support of optical, and electrochemical sensors could be used in the selective, and sensitive determination of transition metals depends on the nature of the modifier. Moreover, l-Arg as an abundant amino acid deserves to perch on the support for optical, and electrochemical determination of transition metals.

## Introduction

**Figure 1:**
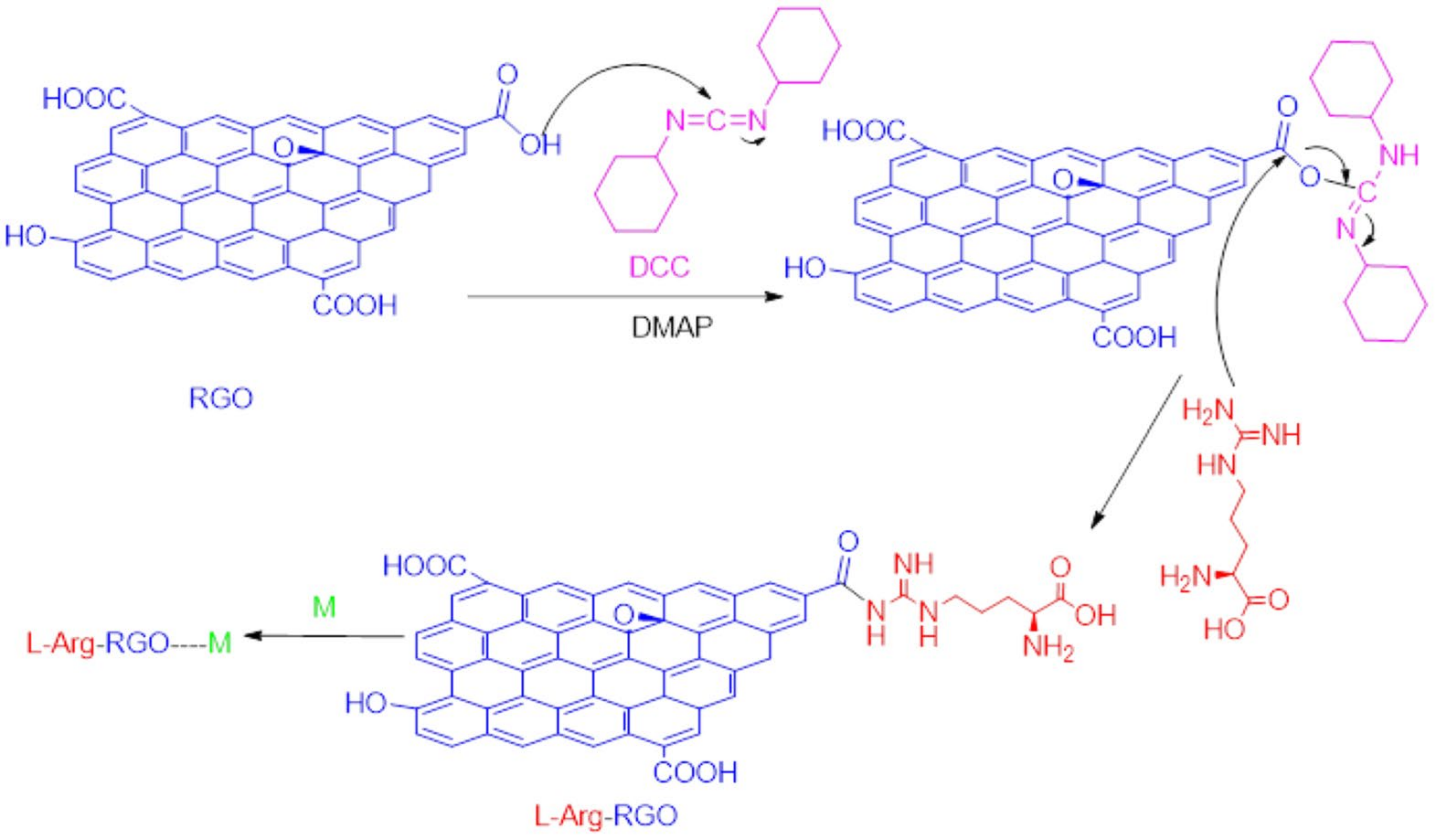
Synthesis of l-Arg-RGO.

Detection and tracking of heavy metals are of significance due to their hazards to the environment and human health^1^. Remarkable number of publications were conducted on the detection of heavy metal ions like Co(II), Cd(II), Cu(II), Hg(II), and Pb(II) by optical and electrochemical approaches^2^. These toxic heavy metals with harmful effects on living organisms are released by human activities in industrial, domestic, and technological applications^3^. Several spectroscopic methods have been utilized to detect and measure small amounts of heavy metals, most of which suffer from expensive instruments, huge arrangements, and highly skilled operators. While optical approaches are fast and facile for determining heavy metals^4,5^, electrochemical techniques have some advantages and benefit from remarkable sensitivity^6–16^. In addition, the inconvenience of storing and moving samples is eliminated using existing portable tools. Anodic stripping voltammetry (ASV) has been reported as a valuable method for determining heavy metals with a lower LOD than other electrochemical methods, by which better results could be achieved using a suitable modifier at the electrode surface^17–19^. Different types of electrodes, and modifiers have been reported to determine the amount of lead using ASV. Nanocomposite-modified electrodes are among the best because of their ability to eliminate memory effects and improve electrode selectivity. Micro-patterned RGO/carbon nanotube/bismuth composite^20^, glutathione-coated magnetic nanoparticles (NPs)^21^, two-dimensional graphitic carbon nitride (g-C_3_N_4_) nanolayers^22^, graphene oxide incorporated mesoporous MnFe_2_O_4_ nanocomposites^23^, 3D honeycomb-like bismuth nanoparticles N-doped carbon nanosheet frameworks^24^, and fluorinated GO^3^ have been used as electrode modifiers to increase selectivity and sensitivity of the sensor. Graphene derivatives are amongst the most widely used modifiers for developing electrochemical sensors^25–35^. Low sensitivity and potential interference of other metals are significant obstacles to the development of graphene-based electrodes in heavy metals detections. Doping graphene nanostructures with heteroatoms, metal or metal oxide NPs, conductive polymers, and active functional groups predominantly solves the issue^3,36–38^. The presence of oxygen-containing functional groups in GO, RGO, and GQD facilitates the functionalization procedure, leading to loading a wide variety of heteroatom-containing compounds on the nanosheets^15,16^. RGO has some advantages over GO that higher conductivity is the most significant^39–42^. Although l-arginine (Arg) has been used to prepare electrochemical sensors^43–47^, this is the first time that RGO was functionalized with l-Arg to be used for a sensitive and selective detection of heavy metals. In the only close report, Verma et al. utilized arginine functionalized magnetic NPs to remove heavy metals from water samples^48^. We believed that l-Arg could be a promising ligand to coordinate heavy metals and provide detectable optical or electrochemical signals. Six heteroatoms on l-Arg create two centers spatially appropriate for anchoring heavy metals (Fig. 1). Furthermore, loading this amino acid on RGO would enhance the optical or electrochemical signals due to the extensive network of free electrons. In this work, for the first time, we functionalized RGO with l-Arg and used the synthesized nanocomposite for the optical and electrochemical detection of heavy metal cations. This nanocomposite was considered for the detection of cations for the following reasons: (1) l-Arg as a multidentate ligand has a promising potential in the complexation with transition metals, (2) l-Arg is an inexpensive and abundant ligand, (3) RGO has a superior surface area providing a tremendous number of sites for the deposition of l-Arg, leading to creation remarkable active centers for the Pb anchoring, (4) RGO has an extended network of sp^2^ carbons, which induces both conductivity and charge stability produced from redox reactions, and (5) RGO is easily accessible and cost-effective material with no toxicity for the environment. We took advantage of the nano-size effect of RGO and the complexation property of l-Arg, which had a synergistic effect on the results.

Amidation reaction was employed to perch l-Arg on the RGO via the reaction between amines and carboxylic acids in the presence of N,N′-dicyclohexyl carbodiimide (DCC) and 4,4′-dimethyl aminopyridine (DMAP) (Fig. 1). DCC/DMAP is a catalytic system to promote the amidation or esterification reactions^49,50^. The obtained results from electrochemical experiments surprisingly showed that contrary to the photometric results, the synthesized sensor selectively detects Pb(II) with high sensitivity.

## Experimental

### Preparation of l-Arg-RGO

Initially, RGO was synthesized by the reaction of graphite (2 g), NaNO_3_ (2 g) and H_2_SO_4_ (90 ml) in an ice bath for 30 min. To the mixture, KMnO_4_ (10 g) was added, and stirring was continued at 50 °C for 2 h. The resultant mixture was treated with deionized water (200 ml) and H_2_O_2_ (12 ml, 35%). Next, the solid was filtered off, washed with HCl (300 ml, 10%). Then, a washing was performed with concentrated HCl (200 ml, 37%) to obtain GO. Finally, the synthesized GO was dried at 120 °C to generate RGO.

For the preparation of l-Arg-RGO, a mixture of RGO (2 g), DCC (0.4 g), DMAP (0.03 g), and DMSO:H_2_O (1:1, 10 mL) was stirred at 60 °C for 1 h. Next, l-Arg was added to the mixture, and stirring was continued for 24 h at 90 °C. l-Arg-RGO was separated via filtration, washed with acetone (3 × 10 mL), and dried in an oven at 60 °C.

Other explanations about materials, methods, and electrochemical parameters are presented in Supplementary Information (SI).

## Results and discussion

### Characterization of l-Arg-RGO

**Figure 2:**
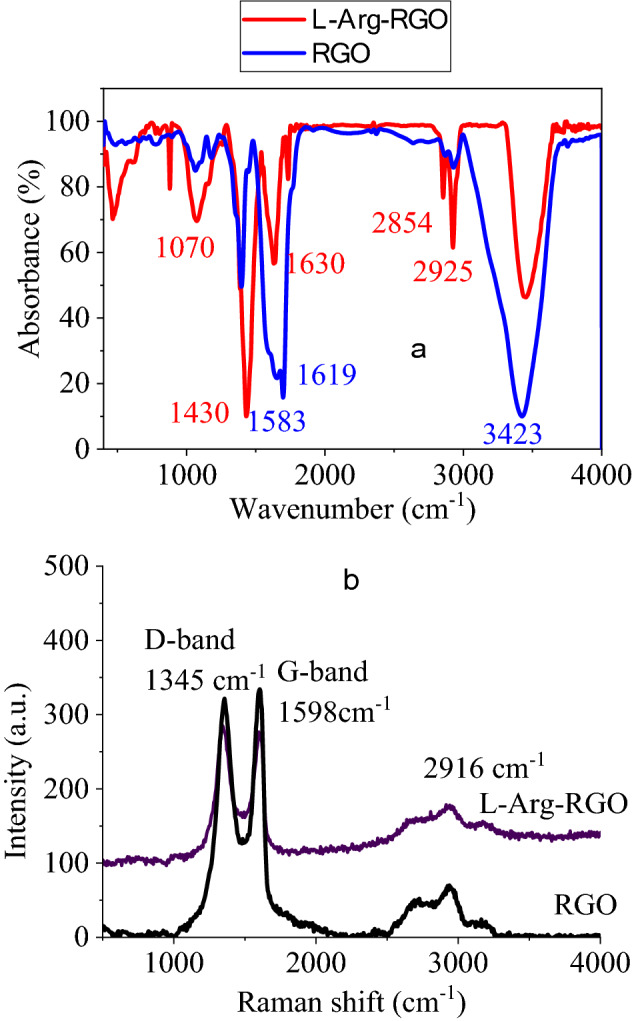
FT-IR spectra (**a**), and Raman spectra (**b**) of RGO and l-Arg-RGO.

Organic transformations owe their progress to spectroscopic analyses, especially FT-IR have played a substantial role in the tracking of functional group addition, elimination, and conversions. FT-IR spectroscopic study was utilized to confirm the reaction between amines of l-Arg and carboxylic acid of RGO (Fig. 2a). The spectrum of RGO indicated sharp peaks related to OH at 3431 cm^−1^, C–H at 2925 and 2854 cm^−1^, C=O at 1630 cm^−1^, and C=C at 1430 cm^−1^. After perching of l-Arg on RGO, the spectrum showed variations in the peaks numbers and wavenumbers, resulting in an amidation reaction. Two significant changes attributed to the modification reaction are the peak shift of C=O from 1630 to 1619 cm^−1^ due to the amidation of carboxylic acids and a sharp peak at 1583 for the N–H bending vibration mode of l-Arg. The repetitious carbon–carbon bonds in graphene derivatives facilitate the spectroscopic studies; in particular, Raman analysis quickly determines this category of materials. Graphene-based compounds could be distinguished by D-band, and G-band ascribed to out-of-plane vibrations of C–H bonds in the defects, and in-plane vibrations of C=C bonds at 1345 cm^−1^, and 1598 cm^−1^, respectively, with I_D_/I_G_ of 1.03 (Fig. 2b).

The Raman spectrum of l-Arg-RGO demonstrated the existence of graphene derivatives in the composite via the appearance of D- and G-bands at 1345 and 1598 cm^−1^, respectively (Fig. 2b). Eminently, the ratio of these peaks’ intensities (I_D_/I_G_) also shed light on the nature of graphene, in which plain graphene reveals a weak peak for D-band (and sometimes no peak) and GO or RGO creates a strong peak as G-band (and sometimes stronger than G-band). A strong peak in the Raman spectrum of l-Arg-RGO showed the RGO nature of the constructed sensor. Meanwhile, the strong D-band indicated the unaggregated sheets, considering graphite has not had this band^51^.

**Figure 3:**
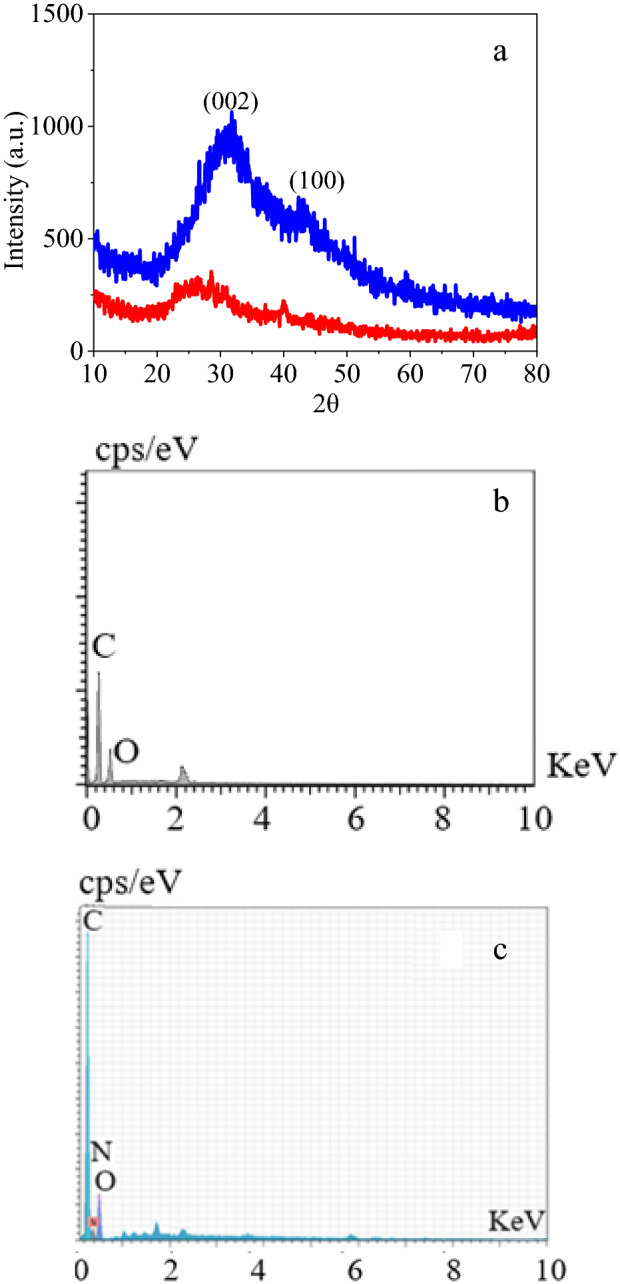
XRD patterns of RGO (red) and l-Arg-RGO (blue) (**a**); EDX spectrum of RGO (**b**), and l-Arg-RGO (**c**).

XRD pattern hints at the crystalline structure of materials with worthy information about the kind of crystals, chemical composition, and physical properties. For the graphene derivatives, this analysis supplies invaluable information about the type of graphene sheets since each graphene, GO, and RGO reveals characteristic peaks. This characterization brought to light a peak at around 2θ = 25° for (002) of RGO and another at about 40° for (001) in the RGO spectrum^52^. The peaks attributed to the RGO also appeared in the l-Arg-RGO spectrum (Fig. 3a). Elemental analysis was conducted on RGO, and l-Arg-RGO by an electron microscope to reveal the constructed atoms in the sensor structure. The presence of C and O atoms in both spectra is ascribed to the graphene nature of the support, in which the low percentage of O atoms indicates RGO structure (Fig. 3b). Moreover, N atoms in the analysis (Fig. 3c) confirmed the loading of l-Arg on the RGO with 1.98 mmol per g of composite (13.87% of N).

**Figure 4:**
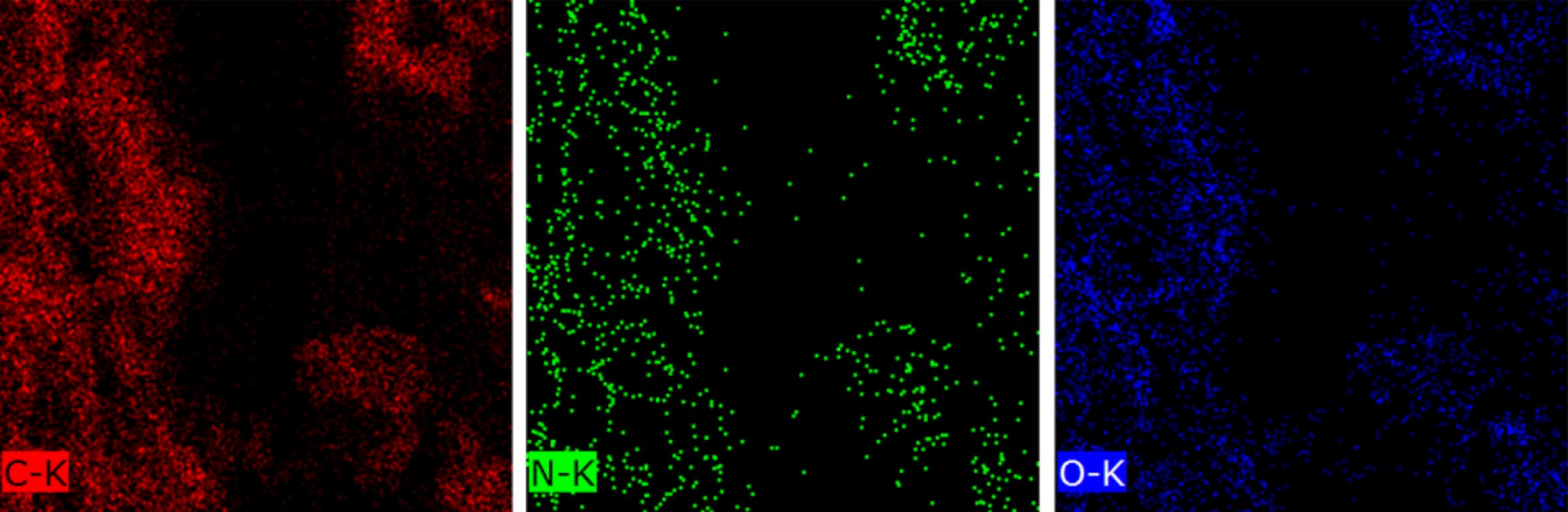
Elemental mapping of l-Arg-RGO.

The emendation of the whole of the RGO sheet was necessary to achieve a uniform sensor and elemental mapping of the sample demonstrated that almost all of the sheet was modified with l-Arg by revealing the nitrogen atoms in all of the sample surface (Fig. 4).

**Figure 5:**
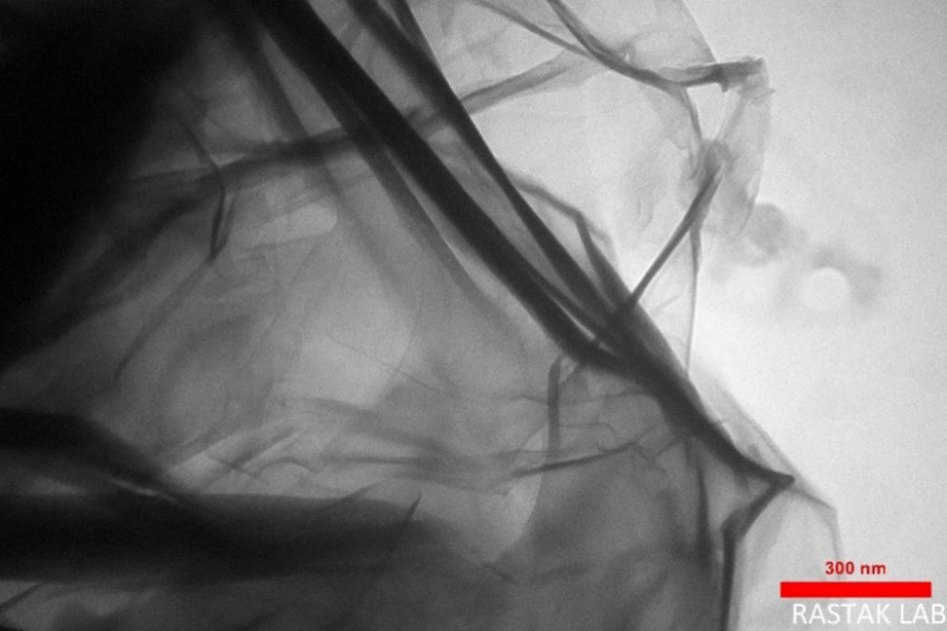
TEM micrographs of l-Arg-RGO.

Transmission electron microscopy micrographs of l-Arg-RGO were prepared to observe the high resolution of RGO sheets (Fig. 5). The image indicated a single-layer sheet with abundant wrinkles. Both the monosheet nature and remarkable wrinkles provide tremendous sites for the actions. Therefore, this analysis suggests that the synthesized sensor has a considerable potential in the detecting metals.

**Figure 6:**
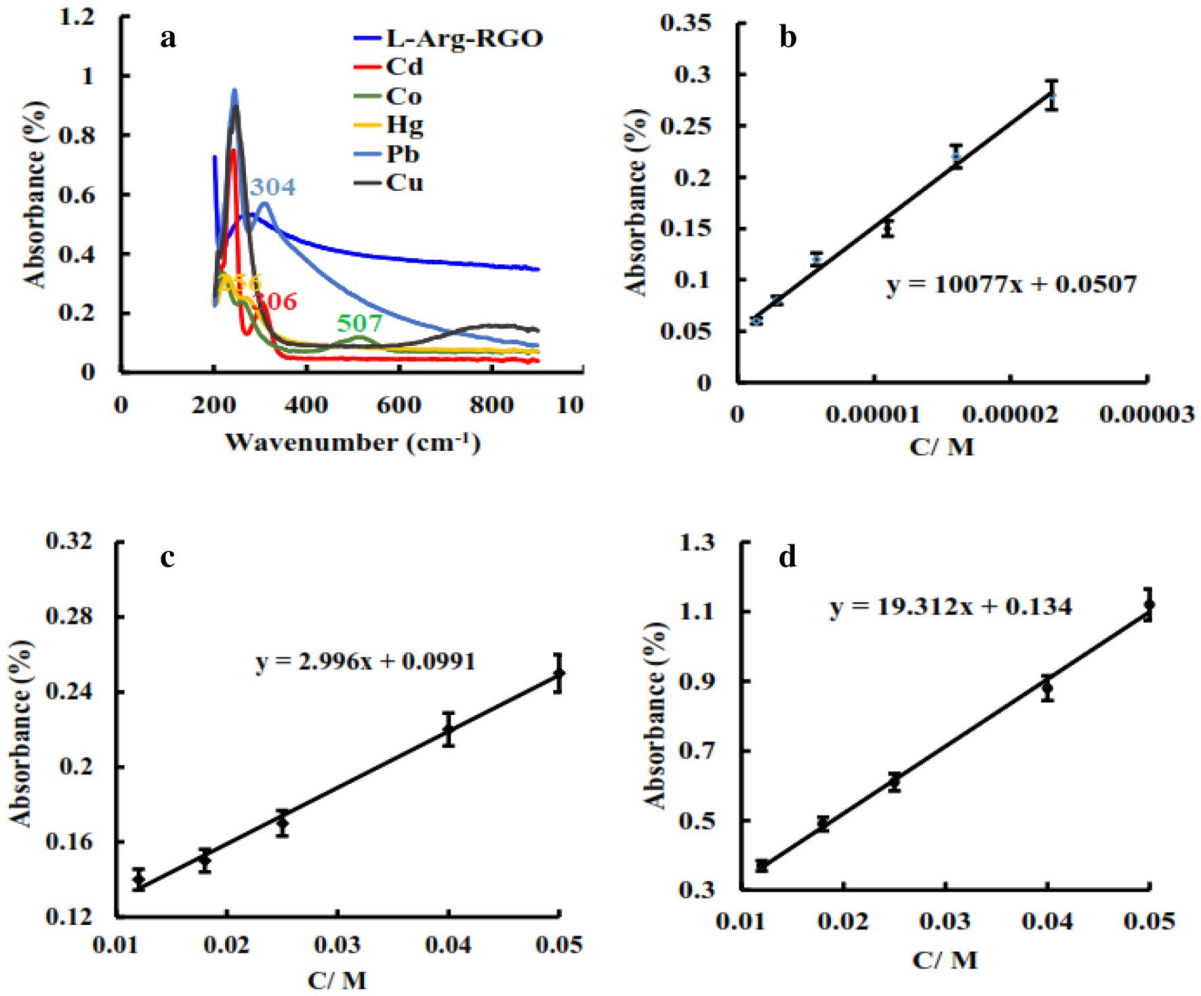
UV–Vis spectra of l-Arg-RGO and its complexes with Cd(II), Co(II), Hg(II), Pb(II), and Cu(II) (**a**), and linear range of Cu(II) (**b**), Co(II) (**c**), and Cd(II) (**d**).

Considering that changes in the optical behavior of the sensor in the presence of a transition metal could provide an easy and fast detection pathway of the metal, the UV–Vis spectra of l-Arg-RGO in some transition metal solutions (0.05 M) were studied (Fig. 6a).

The sensor appeared new absorption peak at 507 nm in the presence of Co(II), which is far from the pure l-Arg-RGO peak at 258 nm. l-Arg-RGO also detected Cd(II), and Pb(II) by the appearance of new absorption peaks at 306, and 304 nm. For Cu(II), a broad peak was observed at > 635 nm when the sample was treated with l-Arg-RGO. The sensor did not show any notable blue shift for the Hg(II), while its detection could be worthy like the previous ones. These cations are the most studied species due to their wide applications and toxicities. Investigation of LOD revealed values of 2.4 × 10^–3^, 1.6 × 10^–2^, and 7.7 × 10^–7^ M for Cd(II), Co(II), and Cu(II), respectively. Recognition of the micromolar of Cu(II) was one of the outstanding results of this study, which attributed to the superior tendency of this cation to amine functionalities. The linear range of the concentration distinguished by absorbance was also surveyed for the cations, in which Cu(II), Cd(II), and Co(II) linear regions were observed (Fig. 6b–d).

### Preliminary experiments

**Figure 7:**
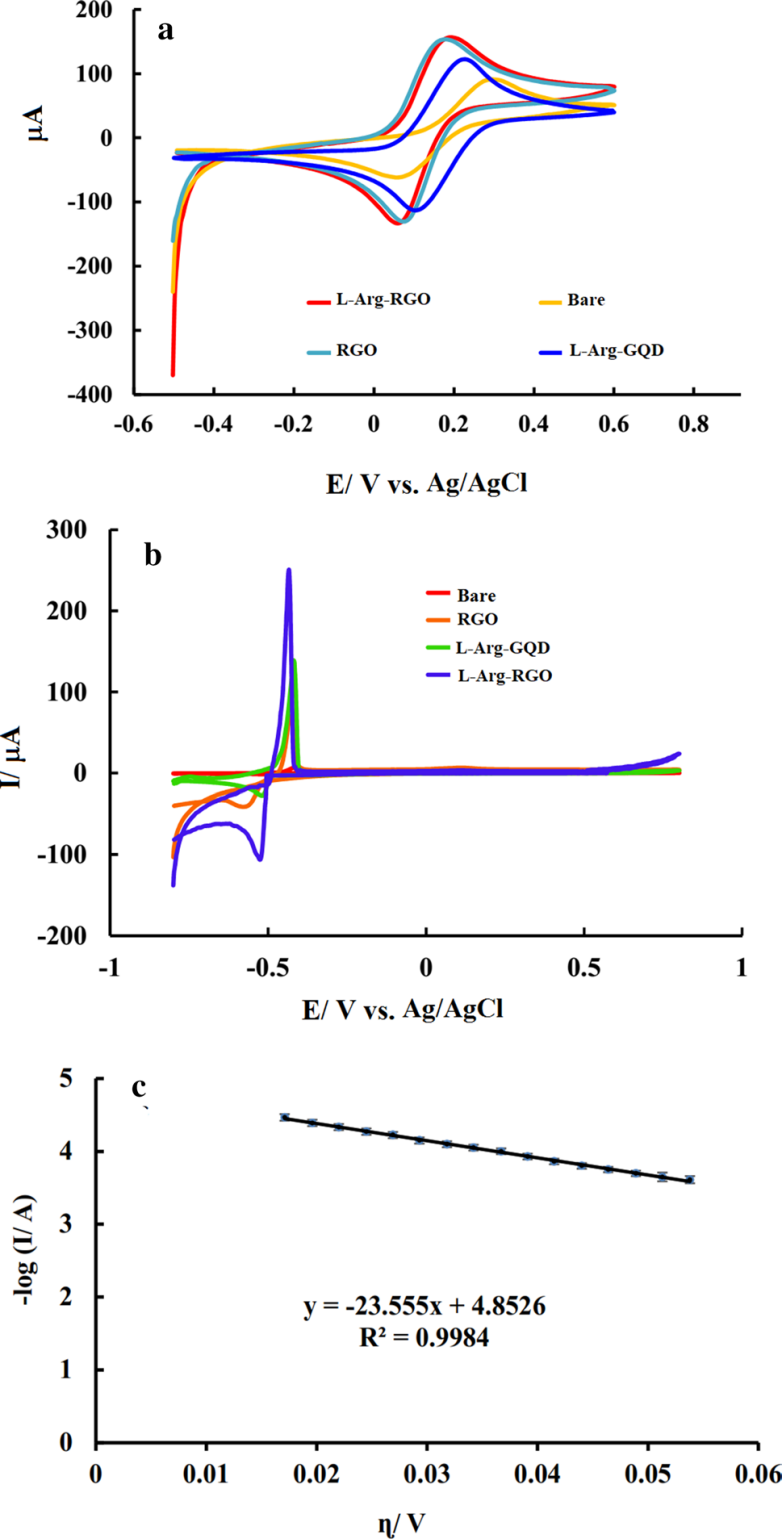
Cyclic voltammograms of bare, RGO, l-Arg-GQD, and l-Arg-RGO modified GCE in 5 mM ${\text{Fe}}({\text{CN}})_{6}^{3 - } {\text{/Fe}}({\text{CN}})_{6}^{4 - }$ solution containing 0.1 M KCl (**a**); Cyclic voltammograms of bare, RGO, l-Arg-GQD, and l-Arg-RGO modified GCE recorded in Pb(II) free KCl solution with pH 6 after preconcentration applying − 0.8 V for 120 s to the electrodes immersed in 2.5 µM Pb(II) with pH 6 (**b**); Tafel plot for anodic branches of the current-overpotential curve obtained from oxidation peak of CV experiment related to l-Arg-RGO (**c**). Scan rate: 100 mV s^−1^.

To evaluate the kinetics of electron transfer and the electrochemically active surface of the bare and modified electrodes with l-Arg-RGO, l-Arg-GQD (for characterization of this nanocompound see SI, Figs. S1–S3), and RGO, cyclic voltammograms were recorded in 5 mM $Fe(CN)_{6}^{3 - } /Fe(CN)_{6}^{4 - }$ solution and compared to each other (Fig. 7a). According to the results, modifying the electrode with all of the modifiers led to the peak current increment and the peak separation decrease, which indicates an increase in the charge transfer rate and the active surface area of the modified electrodes as reported previously based on electrochemical impedance spectroscopy (EIS) experiments^53–56^. Also, based on the results RGO and l-Arg-RGO modified electrodes showed higher current and lower peak separation compared to correspondence species of GQD and l-Arg-GQD which proves better transfer of electrons in RGO-based modifiers.

Numerous electrochemical experiments were conducted using different electrodes to investigate the effect of immobilized l-Arg-RGO on the adsorption of Pb(II) from the solution. The resulting voltammograms at the bare electrode, RGO, l-Arg-RGO, and l-Arg-GQD modified electrodes after 2 min preconcentration applying − 0.8 V as accumulation potential are shown in Fig. 7b. Results demonstrated different behavior for the bare and modified electrodes. No measurable signal was observed at the unmodified GCE electrode. However, a pair of redox peaks appeared on the surface of the modified electrodes, which was related to the oxidation of lead and reduction of the oxidized form. Better results at the electrodes modified by functionalized nanoparticles (l-Arg-RGO and l-Arg-GQD), could be attributed to the superior active surface of the electrode and the ability of the modifier to adsorb Pb(II) cations. In addition, under similar conditions, a better-amplified signal was detected at the l-Arg-RGO/GCE surface in comparison to l-Arg-GQD/GCE, undoubtedly related to better conduction of the nanomaterial and adequate immobilization of the modifier on the GCE. According to Fig. 7b, it can be seen that in cathodic scans, the reduction current grows due to the conversion of Pb(II) cations to metallic lead and metal deposition at potentials less than − 500 mV. The intersection of the anodic and cathodic curves in the cyclic voltammogram confirms the electrochemical perching of lead on the electrode surface. Core nucleation and deposition of metal on the surface of non-metallic electrodes occurs in more negative values than the lone metal because of the inconsistency of the metal and the substrate.

Amino acids tend to adsorb heavy metal ions by functional groups in their structure^48^. During the accumulation step, Pb(II) ions can be gathered on the surface of the electrode due to the interaction with charged or polar functional groups on the amino acid structure, such as hydroxyl, amide, guanidinium, and the formation of an intermolecular bond with axially coordinate or other metal-amino acid species^57^. Meanwhile, applying an appropriate potential to the working electrode in this step accelerate the accumulation process by electrostatics attraction and reduction of the accumulated cations to the metallic lead. The accumulated metal is then oxidized by potential scanning in a positive direction, and a higher oxidation signal appears for the nanostructure-modified electrode.

The diagram of current logarithm versus over-potential variations in CV (Fig. 7c) was used for the kinetics studies and based on the slope of this curve using the following relationship:

$$Slope = \frac{{\left( {1 - \alpha } \right)n_{\alpha } F}}{2.3RT}$$

The value of (1 − α)n_α_ was equal to 1.39, which with assumption 2 for the number of electrons involved in the rate-determining step, α was calculated as 0.31. Also, CV tests using different scan rates showed a linear correlation between scan rate and peak current, which proves the correlation between the peak and the adsorbed species. Using the slope of I_p_ versus *v* applying equation $I_{p} = \frac{{n^{2} F^{2} vA\Gamma^{*} }}{4RT}$, the value of AГ* was calculated to be approximately equal to 0.23 × 10^–9^. The value of log i_0_ determined from the intercept of the plotted curve (Fig. 7c) was about − 4.85 and using the equation: $i_{0} = nFA\Gamma^{*} k^{0}$ by placing known values, standard electron transfer rate constant, k^0^ was calculated to be approximately 0.31 s^−1^.

Chronocoulometry in blank electrolyte and Pb(II) containing solution were done to approve adsorption of the analyte, too. In these experiments, the different intercept from curve Q versus t^1/2^ for these solutions showed that the analyte is adsorbed on the surface of the electrode.

In addition, the electroactive surface areas of different electrodes were calculated using chronocoulometry in 1 mM solution of K_3_[Fe(CN)_6_] and the slope of Q versus t^1/2^ obtained from chronocoulograms based on the Anson equation:

$$Q = \frac{{2nFACD^{1/2} t^{1/2} }}{{\pi^{1/2} }} + Q_{dl} + Q_{ads}$$

Herein, the charge (coulombs), the number of electrons transferred, the real electrochemical surface area of the electrode (cm^2^), Faraday’s constant (96.485 coulombs/mole), the concentration and the diffusion coefficient (cm^2^/s) of the probe denoted as Q, n, A, F, C and D respectively. The real electrochemical surface areas (A) of the bare GCE, RGO/GCE and l-Arg-RGO/GCE were calculated to equal 0.028, 0.078, and 0.072 cm^2^ which is in good agreement with CV results.

### Electrochemical measurement

After optimization of electrode modification (see SI, Figs. S4–S7), differential pulse-anodic stripping voltammetry (DP-ASV) and square wave-anodic stripping voltammetry (SW-ASV) was applied for the determination of trace amounts of lead ions by modified and unmodified electrodes. For this purpose, after Pb(II) accumulation in a stirred solution of 3 nM analyte applying − 0.8 V for 60 s, potential sweeping from − 0.8 to − 0.1 V was done by square wave voltammetry (SWV) or differential pulse voltammetry (DPV) methods in that the accumulated species were stripped from the l-Arg-RGO/GCE surface. The peak shape and current value of the resulting voltammograms showed that DP-ASV is the better choice for performing sensitive lead analysis with the proposed electrode.

**Figure 8:**
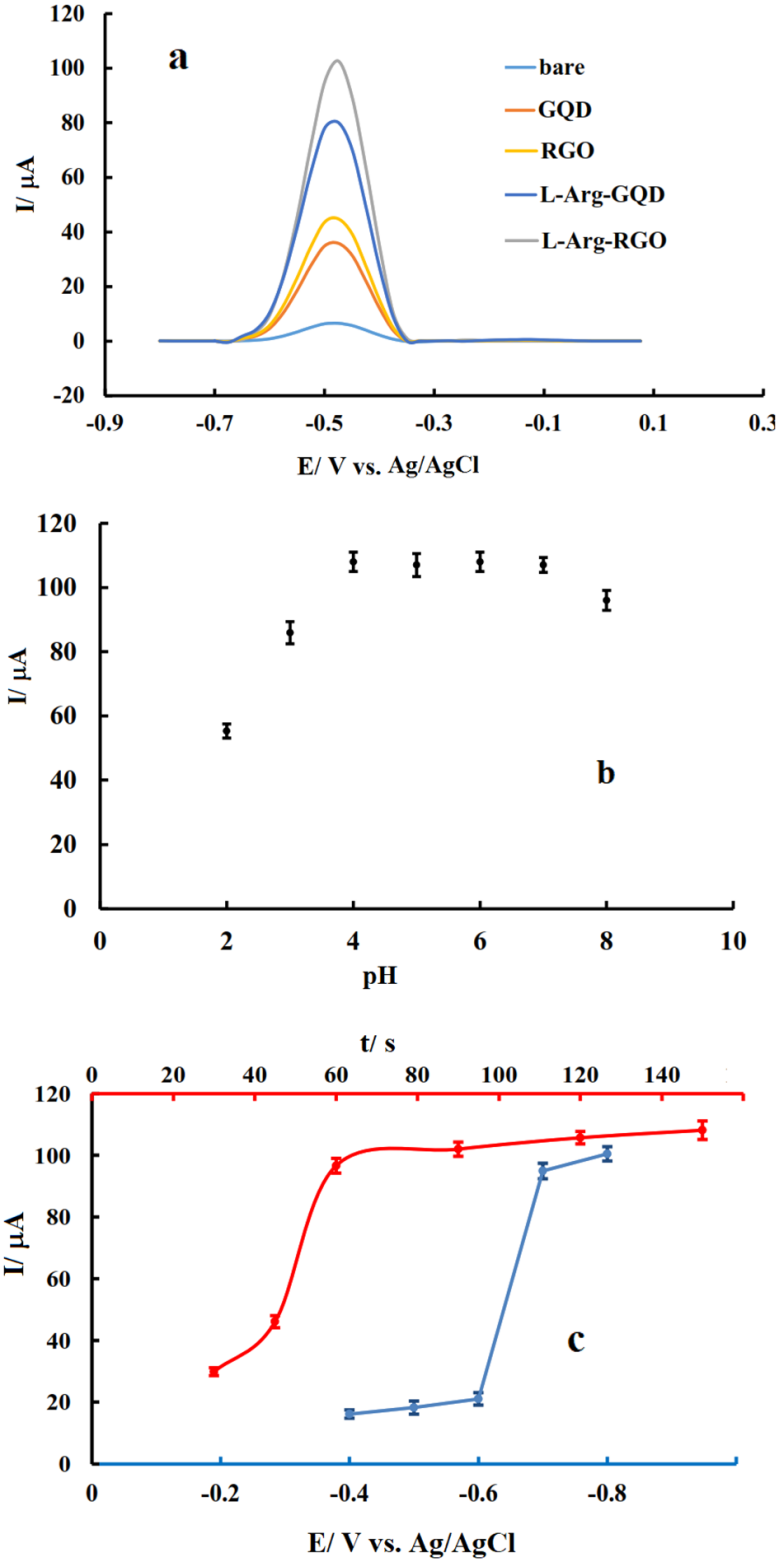
Differential pulse voltammograms of bare and modified GCEs recorded after preconcentration in 3 nM Pb(II) with pH 6 applying − 0.8 V for 60 s (**a**); Variation of DPV signal of l-Arg-RGO/GCE recorded after preconcentration in 3 nM Pb(II) with various pH values applying − 0.8 V for 60 s (**b**), Variation of DPV signal of l-Arg-RGO/GCE recorded after preconcentration in 3 nM Pb(II) with pH 6 applying − 0.8 V for different times (time optimization) or different potentials for 60 s (optimization of potential) (**c**). Scan rate: 0.1 V s^−1^. Stripping condition: Pb(II) free KCl solution with pH 6.

For this experiment, all kinds of electrodes (bare, GQD/GCE, RGO/GCE, l-Arg-GQD/GCE, and l-Arg-RGO/GCE) were put in a stirred solution of Pb(II) applying − 0.8 V for pre-concentration. Then, after washing with deionized water, the electrodes placed in a solution that did not contain lead cation for a DPV experiment. The voltammetric test showed that Pb(II) did not react with anything at the bare electrode with a just tiny peak (approximately 6.5 μA) appeared at this surface (Fig. 8a). After modification of GCE with nanomaterial, the current increased because of the nano-size effect of the modifier which increases surface area and charges transfer rate. The current values related to RGO (44 μA) and l-Arg-RGO (102 μA) are higher than ones at GQD (36 μA) and l-Arg-GQD (80 μA) which may be related to the better functionalization and immobilization of RGO containing compounds. On the other hand, l-Arg functionalized GQD and RGO had better responses compared to non-functionalized ones, which are related to the favorable effect of charged or polar functional groups in the l-Arg structure which result in more adsorption of the analyte.

### Effect of voltammetric variables

The influence of variables such as pH, solution type, accumulation potential, and accumulation time affecting sensor performance was studied. The buffer type and pH of the solution at the accumulation stage are the significant factors affecting the amount of gathered lead and the voltammetric signal. For this study, 0.5 M solutions of KCl, KNO_3_, and PBS (0.1 M phosphate buffer + 0.5 M KCl) as background electrolytes were prepared and used to prepare Pb(II) solutions. Based on gained signals (Fig. S8), the best solution for Pb(II) analysis was KCl according to the obtained voltammetric signal. Consequently, KCl was selected as the appropriate electrolyte for Pb(II) analysis. Multiple solutions using KCl with different pH values having a fixed amount of Pb(II) were prepared and applied as the analyte. Results (Fig. S9) showed that by rising pH up to 4, signals were increased and then leveled off until 7 (Fig. 8b). This increase is ascribed to the availability and freedom of electron donor groups at the modifier structure^48^. Increasing pH to higher values decreases the obtained signal may be due to the competition of hydroxyl ions for interaction with Pb(II). Therefore, KCl with pH = 6 was selected as the optimal accumulation condition for Pb(II) determination by the current electrode.

The stripping solution pH did not influence on the obtained signal (Fig. S10), therefor for simplicity and to avoid solution change, the accumulation condition was applied in the stripping step. It is also worth mentioning that based on the results, chloride ion plays a vital role in the creation of metal-modifier complexes on account of the forming an intermolecular bond with axially coordinate^57^. Similarly, the potential and time of accumulation were among the significant factors affecting the amount of analyte collected at the electrode surface in the stripping analysis. In order to investigate the impact of accumulation potential, after preparing the electrode and placing the electrodes in the electrochemical cell, different pretreatment potentials were applied to the working electrode exposed in the stirred analyte solution, and then stripping was performed after 5 s rest time in the quiet solution (Fig. S11). The variations in the oxidation peak current versus accumulation potential imposing a negative potential to the l-Arg-RGO/GCE, favored the reductive accumulation of the Pb(II) on the electrode, leading to the maximum voltammetric signal for accumulation potential of − 0.8 V (Fig. 8c). To investigate the effect of Pb(II) accumulation time, − 0.8 V as optimum potential was applied to the working electrodes exposed in the stirred analyte solution for different times with the subsequent stripping after 5 s rest time in the quiet solution (Fig. S12). Changes in the oxidation peak current versus accumulation time showed that the best time and maximum voltammetric signal were for the accumulation time of 60 s and remained constant at higher values (Fig. 8c). In other words, it can be concluded that the modifier reaches a saturation state by applying − 0.8 V for 60 s to the l-Arg-RGO/GCE, where these values were selected as the optimum amount for the accumulation step in the following experiments.

### Concentration effect and detection limit

**Figure 9:**
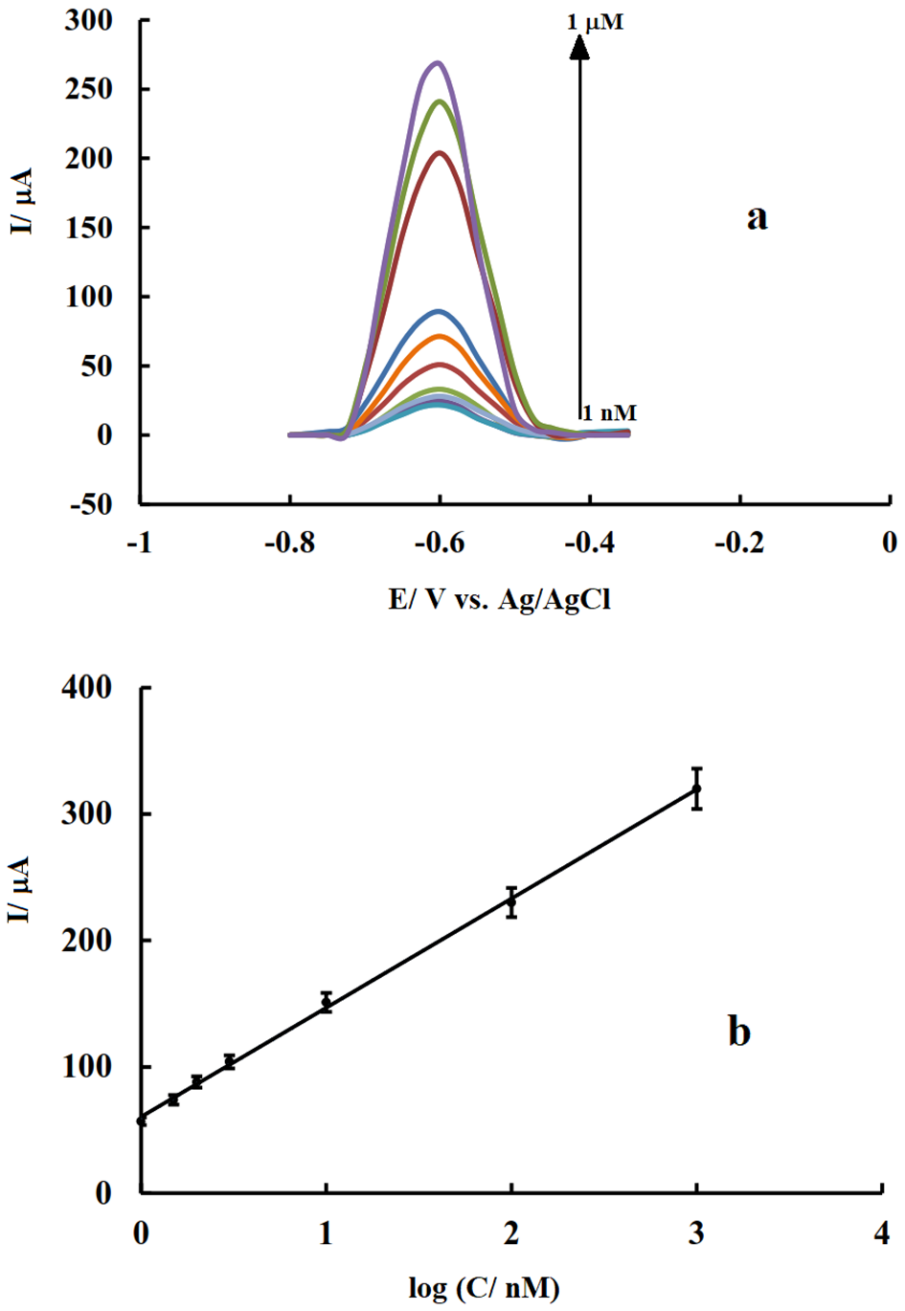
DPV response of l-Arg-RGO/GCE recorded after preconcentration applying − 0.8 V for 60 s to the electrodes immersed in a stirred solution of Pb(II) with different concentration (from 1 nM to 1 μM) (**a**); Calibration curve plotted from presented voltammograms. Accumulation condition: KCl (pH 6) applying − 0.8 V for 60 s; Stripping condition: Pb(II) free KCl solution with pH 6 (**b**); Scan rate: 0.1 V s^−1^, Stripping condition: Pb(II) free KCl solution with pH 6.

By increasing Pb(II) concentration, obtained signals rise and then level off at higher values because of the saturation of the active sites in the modifier structure. To check the effect of concentration and draw a calibration curve in the corresponding linear range, the electrode was floated in the electrolyte solution. By adding different volumes of standard analyte solution, the analyte was accumulated to record the corresponding voltammetric signal after each increment in an optimum experimental condition. The obtained calibration curve (current versus concentration) is shown in Fig. 9.

According to the resulting calibration diagram (Fig. 9), the signal changes in the concentration between 1 nM to 1 μM were linear with a correlation coefficient of 0.998. Considering the minimum concentration that produces a signal equal to three times the standard deviation of the blank sample and using the calibration equation: $y(I/\mu A) = 87.77\log C(C/nM) + 57.52$, LOD was calculated 0.06 nM. The obtained detection limit is comparable to the values reported for other modified electrodes (Table 1). A better LOD can be obtained using a linear calibration curve, but with a limited linear range. The excellent results obtained for the determination of lead ions may be related to the presence of charged or polar functional groups in the modifier structure, appropriate functionalization, and proper stabilization of this novel modifier at the electrode surface.

**Table 1:** Comparison analytical parameters of l-Arg-RGO/GCE with other modified electrodes as Pb(II) sensor.

Modified electrode	Method	Linear range	Detection limit	Ref.
Fe_3_O_4_@SNW1*	SWASV	0.003–0.3 µM	0.95 nM	^16^
Mn-MoS_2_/MWCNTs/NA GCE*	DPASV	0.96–480 nM	0.38 nM	^15^
3D Bi-NCNF*/GCE	SWASV	4.8–579 nM	0.145 nM	^24^
MnO_2_-C/GCE	LSV	10–100 µM	0.027 µM	^58^
Ni based MOF*/GCE	SWASV	0.5–6 µM	0.508 µM	^59^
AuNP/[Ru(NH_3_)_6_]^3+^/Nafion	ASV	1.45–3.62 µM	0.217 µM	^60^
G-PANI-PS/SPCE	SWASV	0.05–2.4 µM	0.016 µM	^61^
NH_2_-MIL-53 (Cr)/GCE	SWASV	0.04–80 µM	0.0305 µM	^62^
Alk-Ti_3_C_2_/GCE*	SWV	0.1–1.5 µM	0.041 µM	^63^
Fe_2_O_3_/Bi_2_O_3_/GCE*	SWASV	0.002–4 μM	0.36 nM	^64^
Bi_2_O_3_/MnO_2_/GO/GCE	SWASV	0.01–10 μM	2 nM	^65^
l-Arg-RGO	DPASV	1 nM to 1 μM	0.06 nM	This work

*Fe_3_O_4_@SNW1: Fe_3_O_4_@Schiff base Network1 modified glassy carbon electrode; Mn-MoS_2_/MWCNTs/NA GCE: Mn-doped MoS_2_/MWCNTs/Nafion modified GCE; 3D Bi-NCNF: 3D honeycomb-like N-doped carbon nanosheet framework decorated with bismuth nanoparticles; MOF: Metal–organic framework; Alk-Ti_3_C_2_: Two-dimensional accordion-like Ti_3_C_2_; Fe_2_O_3_/Bi_2_O_3_*:* shuttle-like α-Fe_2_O_3_ nanoparticles decorated β-Bi_2_O_3_ microspheres.

### Real water sample analysis

The proposed electrode was successfully used to measure lead in tap water (Urmia city) and river water (Liqvan Chay River), which indicates its ability to analyze real water samples. We performed experiments with undiluted tap water and tenfold diluted river water samples by the standard addition method. The absence of an oxidation peak of lead in the results achieved in tap water indicates that the concentration of Pb(II) in this sample is less than the LOD of the proposed sensor. Table 2 reports the results of the analysis of Pb(II) in river water samples. The concentration of lead in river water, taking into account the dilution coefficient, was about 29.5 nM, which is less than the allowable value expressed by WHO (48 nM). Also, the prepared sensor was applied to measure the concentration of Pb(II) in spiked samples (Table 2). These measurements show a recovery of more than 95% and a standard deviation below 5%, which confirms the excellent accuracy and precision of the proposed sensor in practical applications, an allowance for the use of the sensor in Pb(II) determination of environmental samples. Similarly, the results of the *t* test showed that at the confidence level of 95%, there was no notable discrepancy between the found values and added values.

**Table 2:** Obtained results for the determination of Pb^2+^ in real samples.

Sample	Pb^2+^ added (nM)	Pb^2+^ found (nM)	Recovery (%)	RSD (%)^a^
Tap water	0	0		
	5	4.89	97.8	3.7
	10	9.91	99.1	3.3
	20	20.21	101	2.1
River water	0	2.95		2.2
	5	7.82	98.4	3.2
	10	13.08	101	4.1

^a^Relative standard deviation values based on three repetitions.

### Effect of interfering cations

Some similar experiments with other metal cations were done to assess the selectivity of the suggested sensor to the Pb(II). For this purpose, l-Arg-RGO/GCE was subjected to the solutions containing 0.1 μM of Zn(II), Ni(II), Co(II), Cu(II), Hg(II), respectively, and a mixture of these cations plus 3 nM Pb(II). Based on the results, in the presence of mentioned ions, no signal was observed, which could be related to the lack of proper interaction between the modified electrode and these cations in the experimental conditions (Fig. S13). The selectivity of the sensor was also assessed in a sample containing all of the interfering and Pb(II) cations. The results indicated that the presence of interfering cations in the solution did not affected the voltammetric signal of the analyte. This selective response to Pb(II) in electrochemical detection was unexpected which proved the selective adsorption of Pb(II) to the modifier. In general, it can be said that in optimal experimental conditions, the prepared electrochemical sensor had a selective performance and special interaction with Pb(II) ions.

### Reusability of the electrodes

To evaluate the repeatability of the sensor, five measurements were performed using the prepared electrode. To reuse the prepared electrode, desorption of the adsorbed cation from the electrode surface is necessary. Our results showed that immersing the modified electrode in 0.1 M EDTA stirred solution could desorb Pb(II) from the electrode surface and recycle the used electrode six times with the relative standard deviation (RSD) of 4.54% for the results of this recycled electrode (Fig. S14). The fast and simple preparation and measurement process with this sensor, along with the 6 times reusability of the electrode hinted at the excellent performance of the proposed sensor. The relative standard deviation over five independently l-Arg-RGO modified electrodes used for the determination of 3 nM of Pb(II) was 3.39%, indicating a remarkable reproducibility of the proposed sensor (Fig. S15). To evaluate the stability of the l-Arg-RGO sensor, the prepared electrode was stored at 4 °C and used on different days (first, second, and so on) after preparation to measure lead. The results revealed that (Fig. S16) after 13 days, the electrode has 95% of its initial response and is stable for 13 days. Due to the simple synthesis of this electrode, 13 days is considered a satisfying duration for the present sensor.

## Conclusions

In this study, RGO was successfully functionalized with l-arginine to afford an optical and electrochemical sensor by a simple strategy. The reaction was proceeded by an amidation reaction of l-Arg’s amines and RGO’s carboxylic acids, in which the inexpensive nature of the starting materials offers synthesis of a cost-effective sensor. l-Arg-RGO successfully identified Co(II), Cd(II), Pb(II), and Cu(II) cations using UV–Vis spectroscopy, in which characteristic peaks appeared in the spectra of each of the mentioned cations far from the peak of the l-Arg-RGO. The abundance of functional groups with appropriate spatial arrangement leads to the easy complexation of the sensor with cations. Moreover, the wide π-network of RGO creates an opportunity for the optical study of the generated complexes. Also, an electrode modified by l-Arg-RGO demonstrated excellent electrochemical behavior in the adsorption and determination of Pb(II) in low concentrations. The presence of charged or polar functional groups such as hydroxyl, amide, and guanidine in the structure of l-Arg increases the affinity of this modifier to Pb(II) ions over a wide range of pH values, and the high adsorption capacity of the modifier makes l-Arg-RGO an excellent modifier in stripping voltammetry experiments. The proposed sensor was utilized for the sensitive and accurate determination of Pb(II) concentration in real water samples by DP-ASV.

## Supplementary Information

Supplementary Information.

## Data Availability

All data generated or analysed during this study are included in this published article and its supplementary information files.
