# Supplementary material for: An optical and electrochemical sensor based on l-arginine functionalized reduced graphene oxide
Source: Sci Rep. 2022 Nov 12;12:19398. doi: 10.1038/s41598-022-23949-5 (PMC9653396; doi:10.1038/s41598-022-23949-5)
Supplement: Supplementary file 1 — Supplementary Information. [file 41598_2022_23949_MOESM1_ESM.docx]

**An optical and electrochemical sensor based on L-Arginine functionalized reduced graphene oxide**

Sanaz Ghanbari^a^, Fatemeh Ahour^a,b*^, and Sajjad Keshipour^a,b^

*^a^Nanotechnology Research Group, Faculty of Science, Urmia University, Urmia, Iran*

*^b^Department of Nanochemistry, Nanotechnology Research Center, Urmia University, Urmia, Iran*

**Email:* [*F.ahour@urmia.ac.ir*](mailto:F.ahour@urmia.ac.ir)

## **EXPERIMENTAL**

## **Materials and methods**

Deionized water was used to prepare the solutions. All chemicals were purchased from Sigma-Aldrich Company. Analytical solutions were prepared using metal ion nitrate salts. High purity graphite powder (Albany graphite deposit) was used in modifier preparation. IR spectra were recorded by the Nicolet FT-IR NEXUS 670 spectrometer (Thermo Scientific, USA) and used to identify the presence of functional groups in synthesized compounds. HANDHELD RAMAN ANALYZER of RIGAKU (FIRSTGUARD model) was used for Raman spectroscopy. TESCAN MIRA III scanning electron microscope and Zeiss Libra transmission electron microscope (working at 100 kV) were used to record FE-SEM and TEM images respectively. XRD instrument of a Bruker D8 ADVANCE X-ray diffractometer was used with a Cu-K_α_ radiation source (λ = 1.5406 Å) operating at 40 kV, 40 mA, and a scanning range of 10–80° 2θ, with a 2θ scan step of 0.015° and a step time of 0.2 second. UV-Vis spectra were recorded by WPA Biowave LifeScience UV-Vis spectrometer using quartz cuvettes with a path-length of 10 mm in H_2_O as the solvent. pH measurements were performed using a digital pH meter (HANNA 212). Ultrasonic bath (KODO model JAC1002) was used to clean the surface of GCE and prepare homogeneous suspensions from modifiers.

## **Electrochemical measurements**

All electrochemical measurements were performed in AUTOLAB PGSTAT 30 equipment. Cyclic voltammetry (CV) and square wave voltammetry (SWV) experiments were performed using an Autolab electrochemical workstation. The electrochemical cell consists of three electrodes containing modified GCE (2 mm diameter) as working electrode, Ag /AgCl (1 M KCl) as reference electrode, and platinum wire as the auxiliary electrode.

The Fe(CN)_6_^3-^/Fe(CN)_6_^4-^ was used as a redox probe to evaluate the surface changes and modifier immobilization at the electrode surface.

To study the electrochemical behavior of modified electrodes in Pb(II) analysis, 5 mL 0.1 M KCl (pH of 6.0) containing an appropriate amount of Pb(II) standard solution was added into the electrochemical cell and then the three-electrode system was put in it. The CVs were recorded in the potential range from -0.8 to 0.8 V with a sweep rate of 100 mV s^-1^. DPV experiments of the target metal ions were recorded in the potential range from -0.8 to 0.1 V with a pulse amplitude of 50 mV. Two steps were involved in the heavy metal ion sensing: (i) deposition of metal ions at -0.8 V for 120 s; and (ii) DP-ASV or SW-ASV conducted from -0.8 to -0.1 V. Square wave anodic stripping voltammetry (SW-ASV) was performed from -0.8 to 0.1 V at a frequency of 25 Hz, an amplitude of 50 mV, a step length of 4 mV and a sweep rate of 0.1 V s^–1^. Prior to each new measurement, the electrode was cleaned by immersing the modified electrode in 0.1 M EDTA stirred solution which could desorb Pb(II) from the electrode surface.

**Modifier preparation**

The modifier was prepared by dispersing 10 mg of prepared L-Arg-RGO in 10 mL 0.1M KCl solution using the ultrasonic bath for 5 min to form a homogeneous solution and then applied for the preparation of working electrode. Before modification, the bare GCE was mechanically polished to a mirror-like surface with a 0.3, and 0.05 µm alumina slurry, then sonicated successively in deionized H_2_O and washed thoroughly with deionized water. Next, L-Arg-RGO was deposited onto the treated electrode surface using an electrochemical platform by performing 70 repetitive potential cycles in modifier solution. The potential range for electrode modification was from -1 to 1 V with a sweep rate of 100 mV s^-1^. After the preparation of modified GCE, it was rinsed thoroughly with deionized water. For comparison, Arg-GQD/GCE was prepared under the same condition. Also, L-Arg-RGO/GCE and L-Arg-GQD/GCE were prepared by casting 3 µL (1 mg mL^-1^) of modifier suspension on the electrode surface and drying at an oven (50 °C). After washing the prepared modified electrodes, we put them in the refrigerator until use.

**Results and discussion**

## **Characterization of L-Arg-GQD**

Comparison of GQD and L-Arg-GQD FT-IR spectra showed that peaks of carbonyl groups at 1705 and 1773 cm-1 were moved to 1579 and 1627 cm-1 due to the transformation of carboxylic acids to amide groups (Fig. S1).

**Fig. S1.** FT-IR spectra of GQD and L-Arg-GQD

XRD pattern of L-Arg-GQD revealed the peak of (002) for GQD (Fig. S2)

**Fig. S2.** XRD diffractogram of L-Arg-GQD

EDX analysis of L-Arg-GQD indicated N atoms attribbuted to the L-Arg in the sensor structure. Also, elemental mapping of N demonstrated homogeneous distribution of this atom in the strcture (Fig. S3)


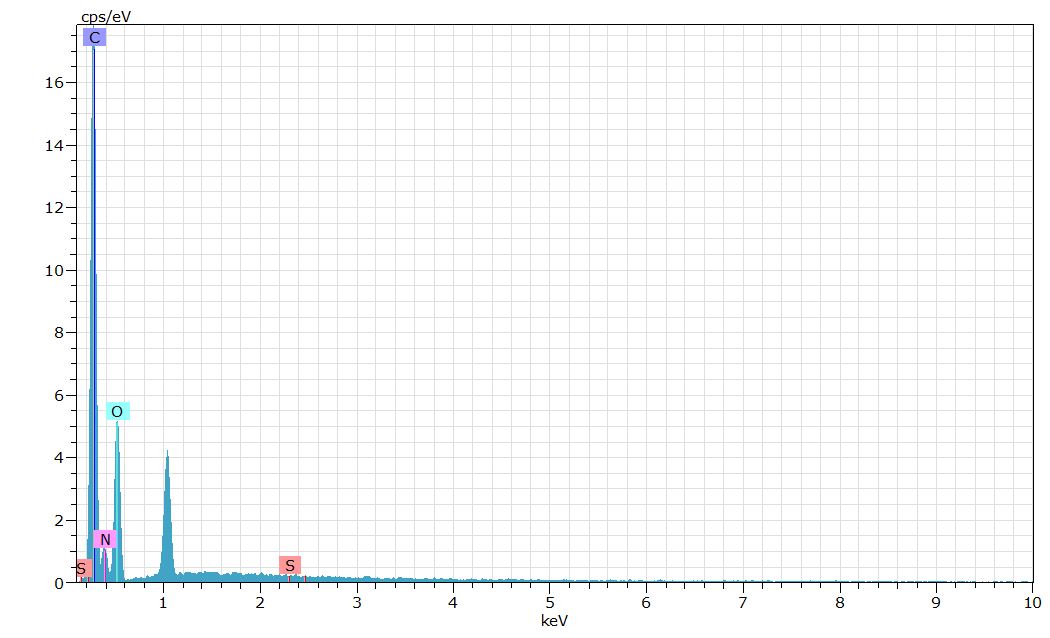


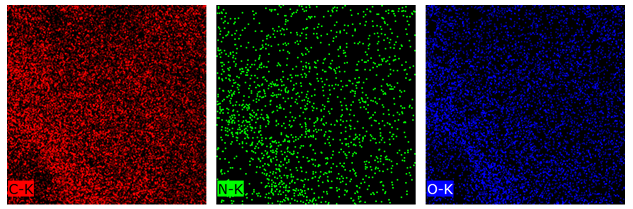


**Fig. S3.** EDX and elemntal mapping of L-Arg-GQD

# Optimization of electrode modification parameters

The first step in preparing a sensor is the modifier immobilization on the surface of the electrode, in which the modifier amount on the electrode surface affect the electrochemical behavior and voltammetric results. In order to evaluate the necessity of pre-concentration, after preparation of the electrode L-Arg-RGO, Pb(II) was measured before and after applying accumulation time, revealing that gathering of Pb(II) for a distinct time on the electrode surface affords better results (Fig. S4). In continue, to select the appropriate modification method, the electrode was modified by two methods of electrochemical and dropping. Results of the Pb(II) measurement by these electrodes demonstrated that electrochemically composed electrodes had better stability with a greater peak current for Pb(II) (Fig. S5). Therefore, electrode modified by electrochemical route was considered for further studies. The main parameters in CV experiments are potential window, sweep rate, and scan number that could increase the sensitivity of the sensor by influencing the quantity of immobilized modifier. It was found that the best potential range for immobilization of modifier was from 0 to 1 V to generate the best Pb(II) oxidation signal. Better accumulation of modifier in this potential range may be related to the electrostatic interactions between negatively charged L-Arg-GO and electrode surface. Also, the lead oxidation signal was enhanced as the number of cycles increased up to 70 cycles with subsequent gradual decrease after that (Fig. S6). This behavior is ascribed to the increment of the modifier amount and resultant surface functional groups up to 70 cycles, and the decrease of conductivity at higher scan numbers. Thus, modifications were done using 70 cycles as the optimum amount. Influence of the potential sweep rate of the modification step was also explored from the obtained signal for lead oxidation. To perform this study, the GCE was pretreated applying different sweep rates in the modification step. Increasing the potential sweep rate up to 50 mV s^-1^ led to the improvement in the immobilization of the modifier at the electrode surface, and signal. But increasing to higher values ​​did not have a positive effect on the Pb(II) voltammetric results (Fig. S7). It can be explained that the lower signals at the electrodes modified applying low sweep rate is related to the decreased conductivity of the electrode due to the long-time duration of the electrode modification and compaction of the modifier layer. Conversely, at high sweep rates, there is not enough time to immobilize a suitable amount of modifier, leading to decreased signal. Thus, 50 mV s^-1^ was chosen as the optimized sweep rate in electrode modification. After optimization of electrode preparation parameters, the prepared electrode used for the sensitive determination of Pb(II).


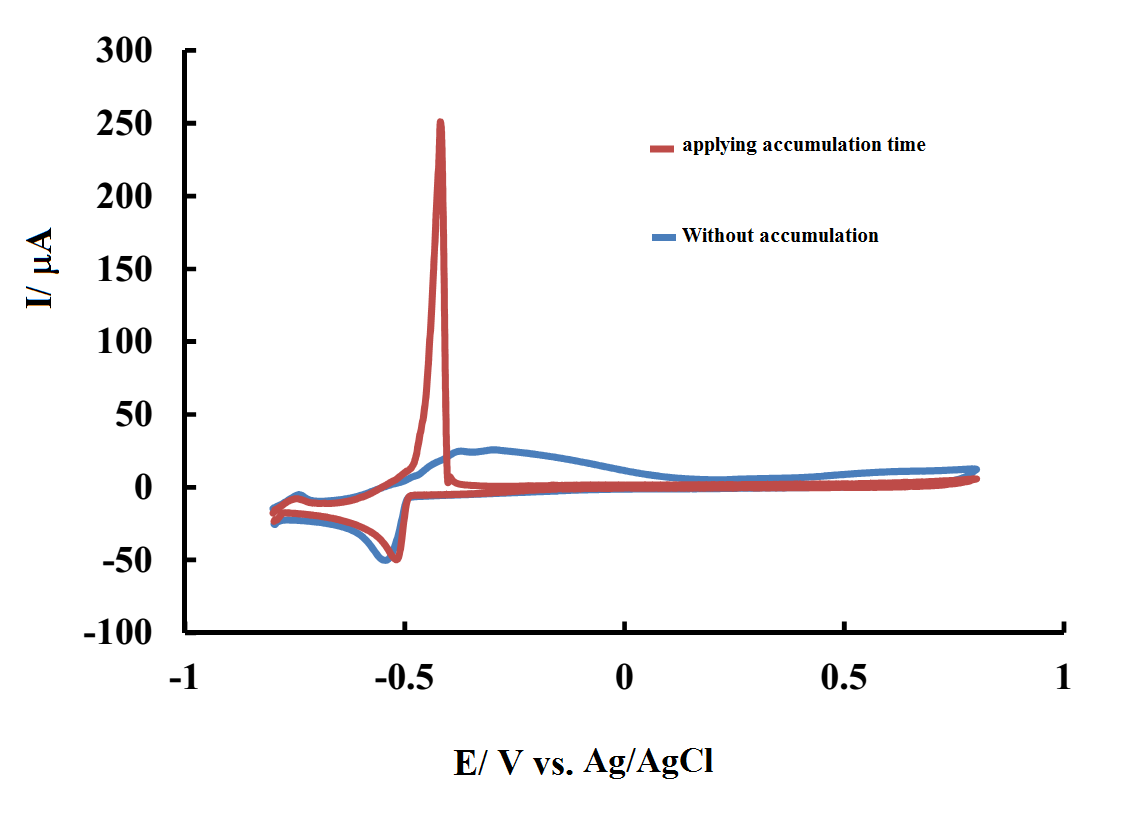


**Fig. S4.** Cyclic voltammograms of L-Arg-RGO/GCE immersed in 2.5 μM Pb(II) solution without accumulation and after applying accumulation time


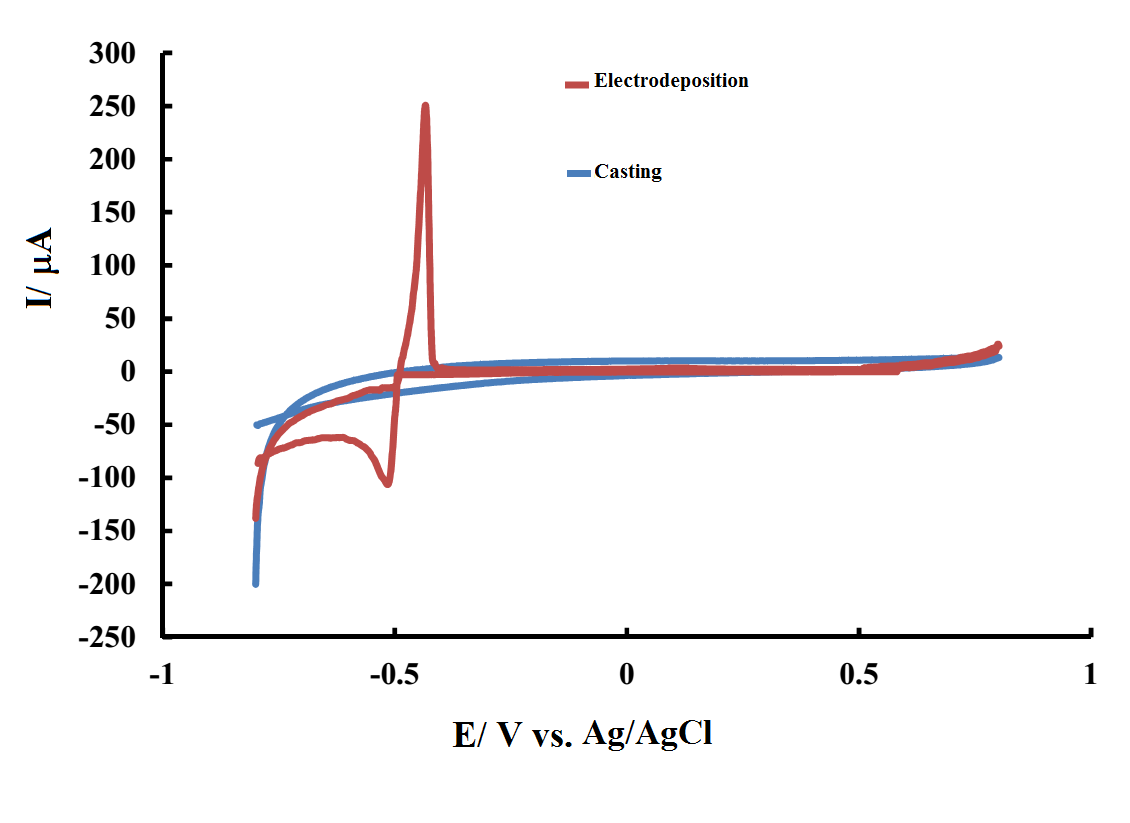


**Fig. S5.** Cyclic voltammograms of L-Arg-RGO/GCE prepared electrochemicaally or by casting immersed in 2.5 μM Pb(II) solution after applying accumulation time


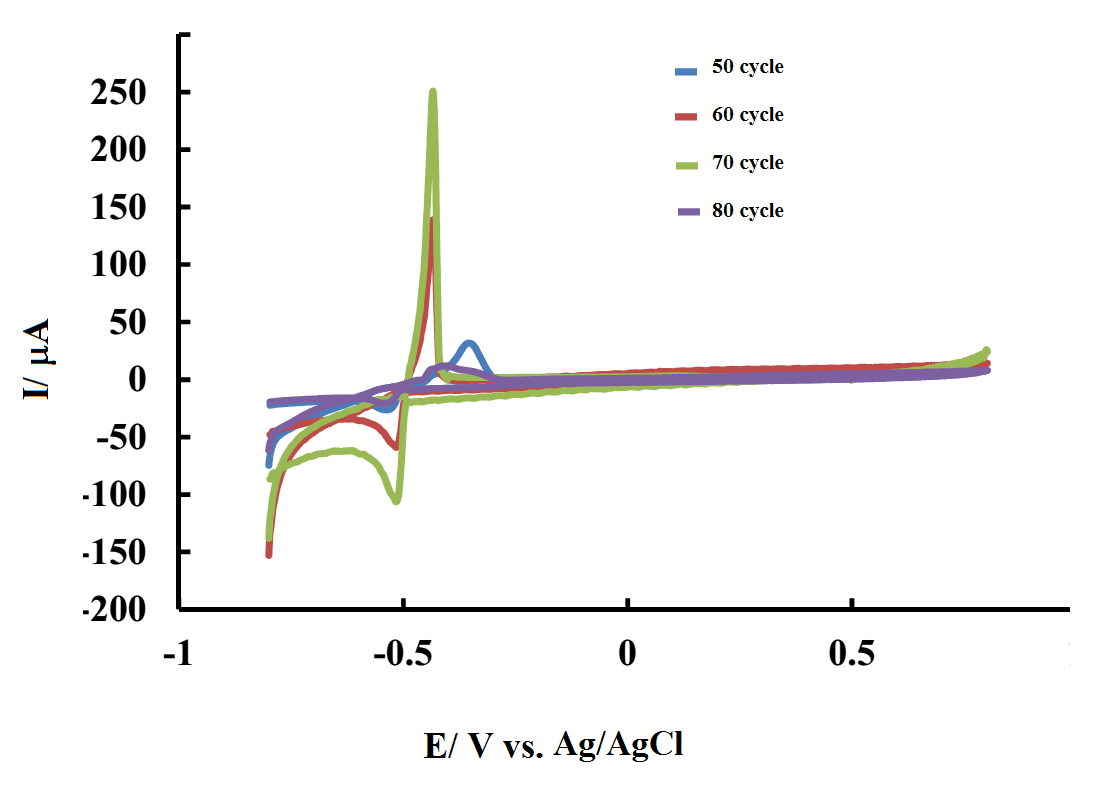


**Fig. S6.** cyclic voltammograms of L-Arg-RGO/GCE prepared electrochemically using different scan numbers after accumulation of 2.5 μM Pb(II) applying -0.8 V for 60 s and stripping in KCl


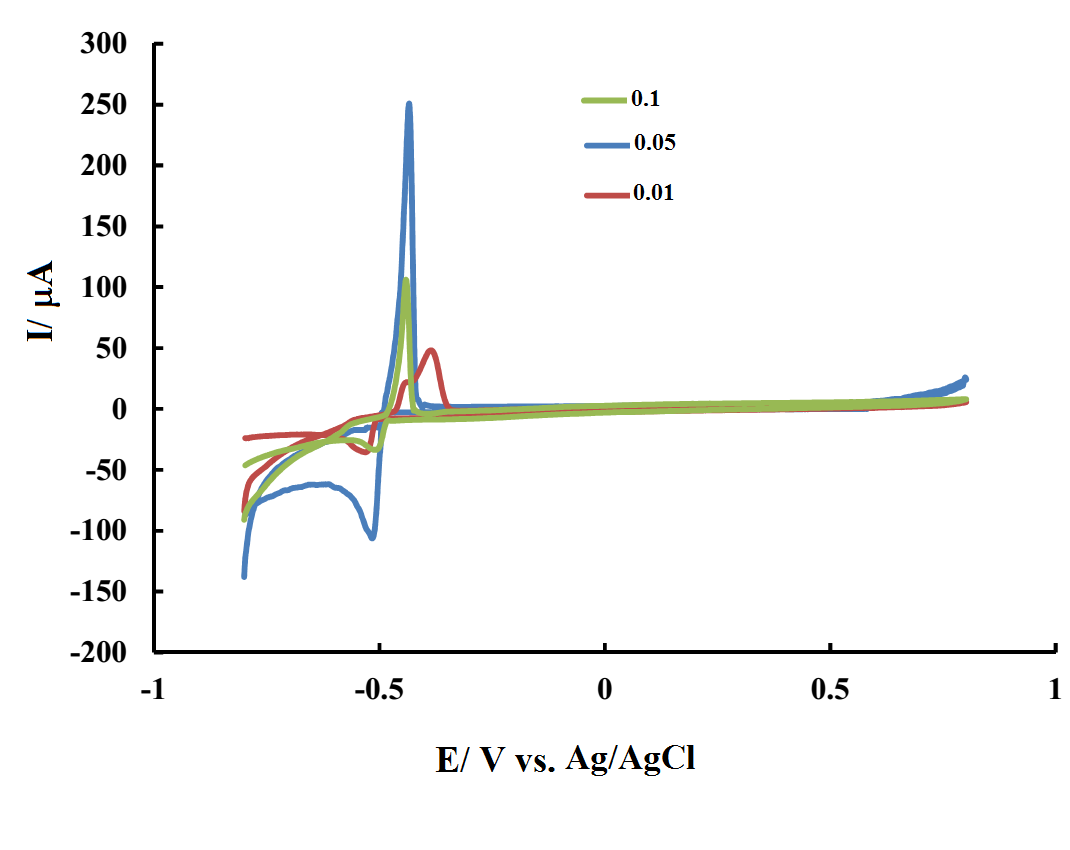


**Fig. S7.** cyclic voltammograms of L-Arg-RGO/GCE prepared electrochemically using 70 scan by different scan rates after accumulation of 2.5 μM Pb(II) applying -0.8 V for 60 s and stripping in KCl.

**Effect of voltammetric variables**


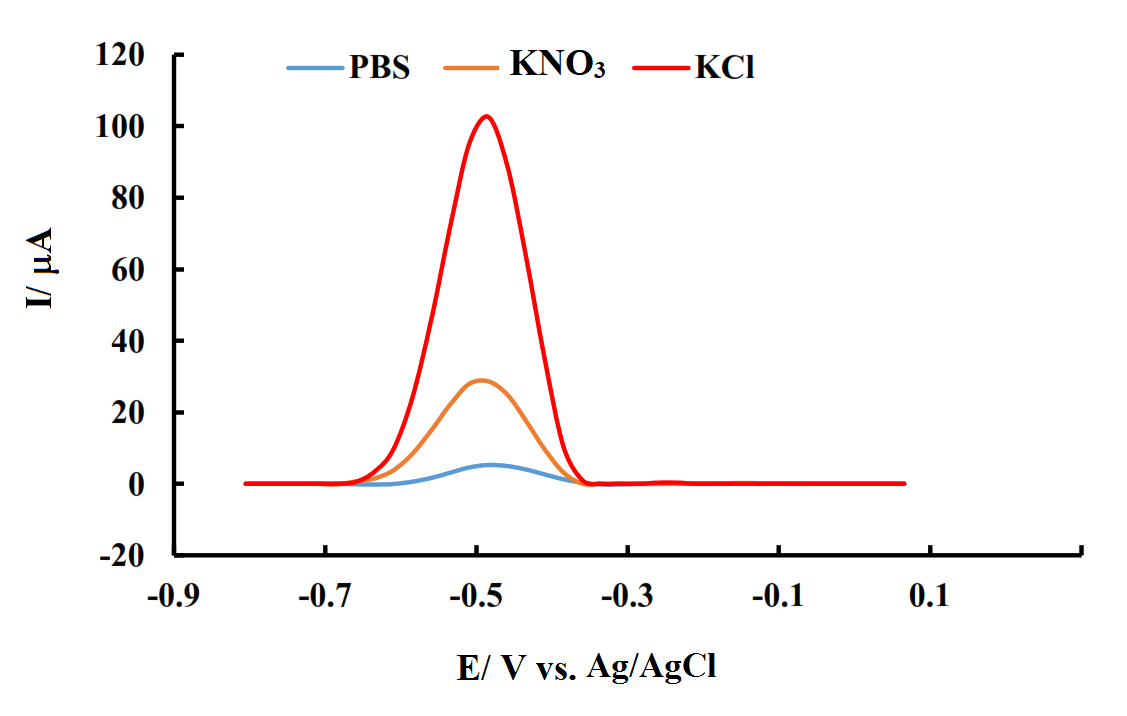


**Fig. S8.** Differential pulse voltammograms of L-Arg-RGO/GCE after accumulation of 3 nM Pb(II) in different buffers applying -0.8 V for 60 s and stripping in KCl.


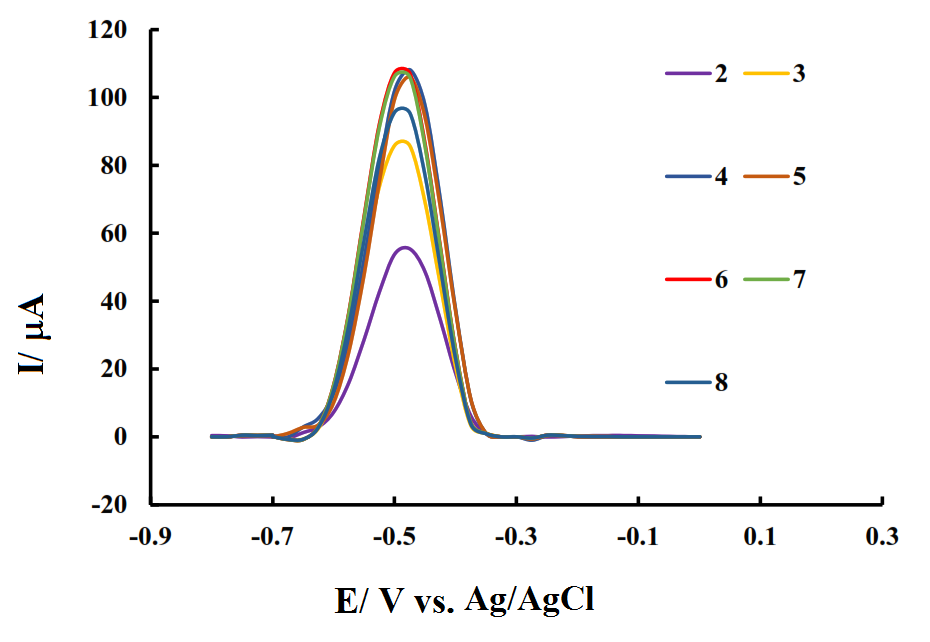


**Fig. S9.** Differential pulse voltammograms of L-Arg-RGO/GCE recorded after dipping in 3 nM Pb(II) with various pH values. Accumulation condition: KCl (pH 6) applying -0.8 V for 60 s


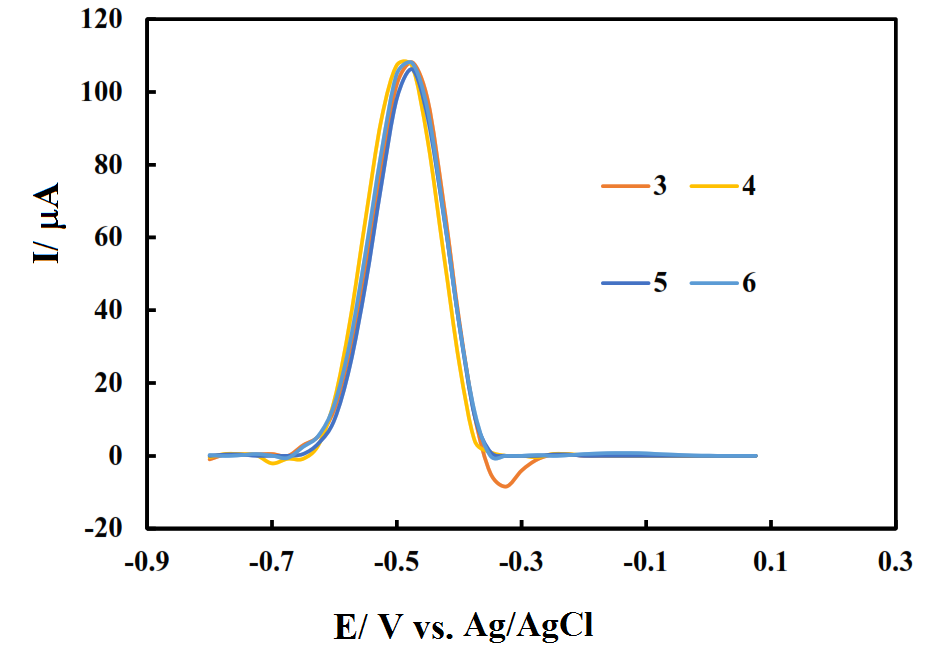


**Fig. S10**. Differential pulse voltammograms of L-Arg-RGO/GCE after accumulation of 3 nM Pb(II) applying -0.8 V for 60 s and stripping in KCl with different pH values.

**
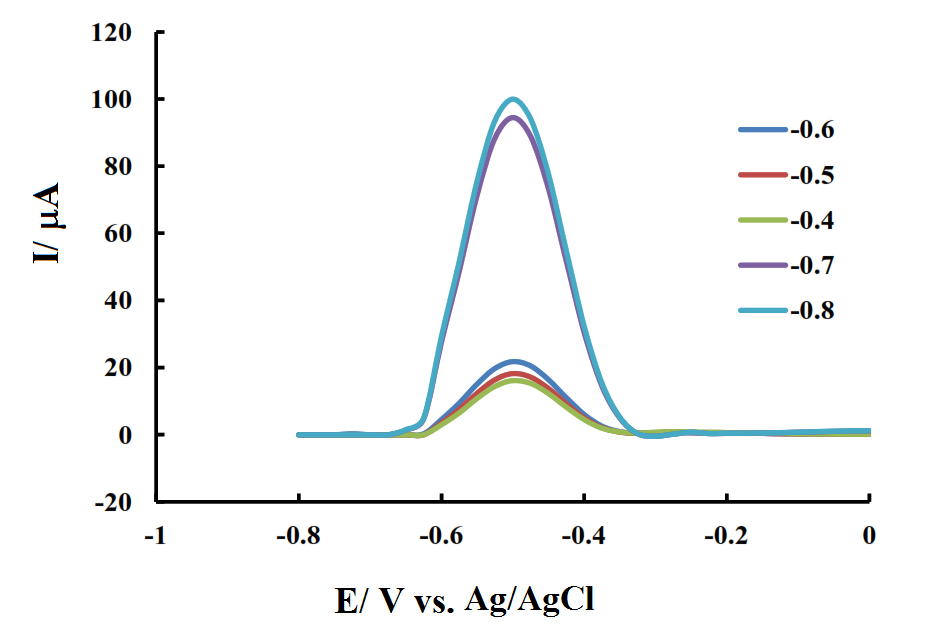
**

**Fig. S11**. DPV responses of L-Arg-RGO/GCE dipped in 3 nM Pb(II) after accumulation applying different accumulation potential. Accumulation conditions: KCl (pH 6) applying different potentials for 60 s (optimization of potential).


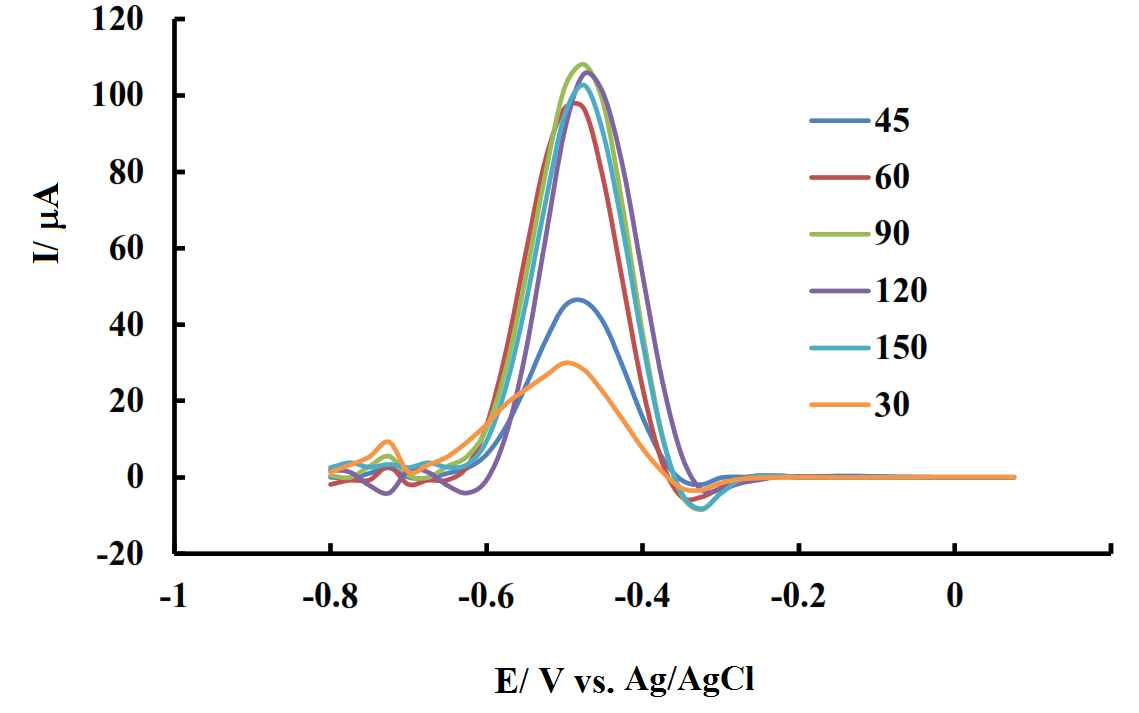


**Fig. S12**. DPV responses of L-Arg-RGO/GCE dipped in 3 nM Pb(II) after accumulation applying -0.8 V for different time intervals. Accumulation conditions: KCl (pH 6).

**Effect of interfering cations**


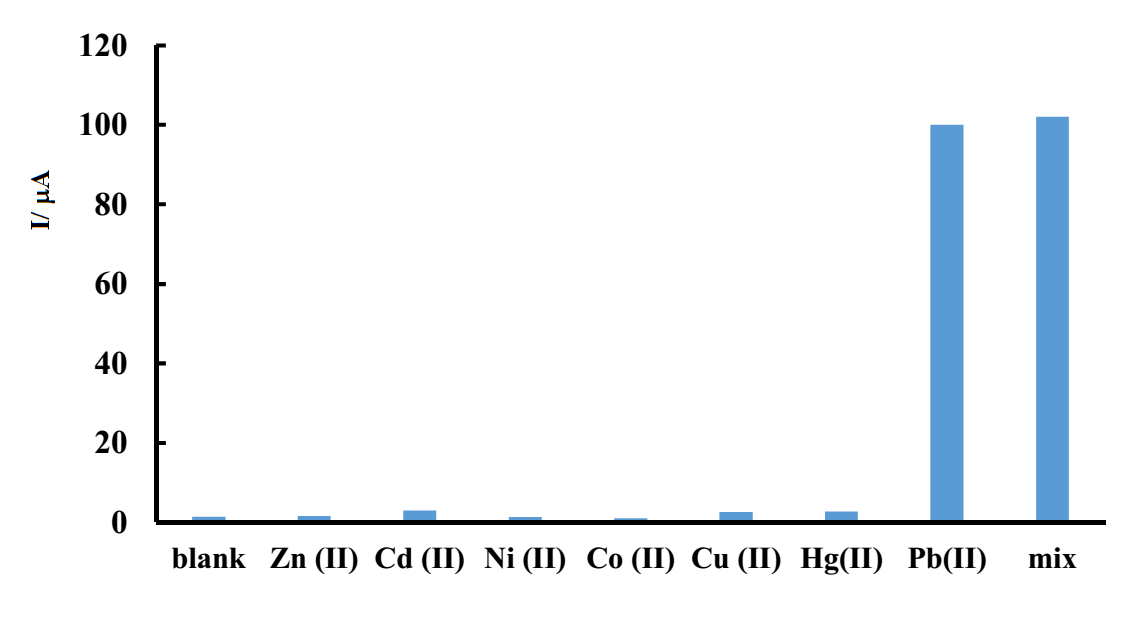


**Fig. S13.** Variation of DPV signal of L-Arg-RGO/GCE dipped in the solutions containing 100 nM of Zn(II), Cd(II), Ni(II), Co(II), Cu(II), Hg(II), and 3 nM of Pb(II) and a mixture of these cations.

## **Reusability of the electrodes**


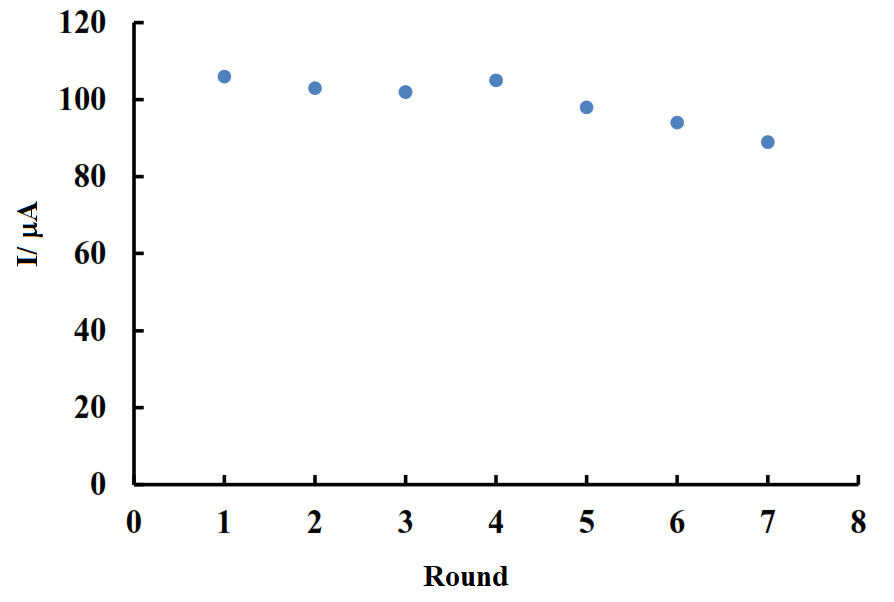


**Fig. S14.** Variation of DPV signal of L-Arg-RGO/GCE dipped in the solutions containing 3 nM of Pb(II) after regeneration in different rounds.

**
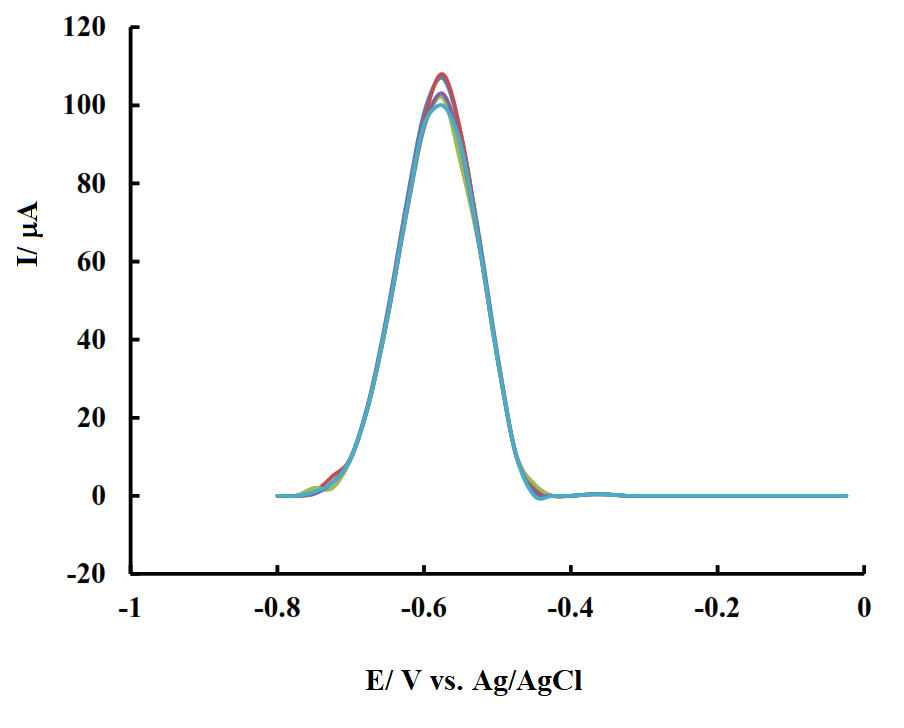
**

**Fig. S15.** DPV responses of five independently prepared L-Arg-RGO modified electrodes in Pb(II) free KCl solution after accumulation applying -0.8 V for 60 s in 3 nM Pb(II). Accumulation conditions: KCl (pH 6).


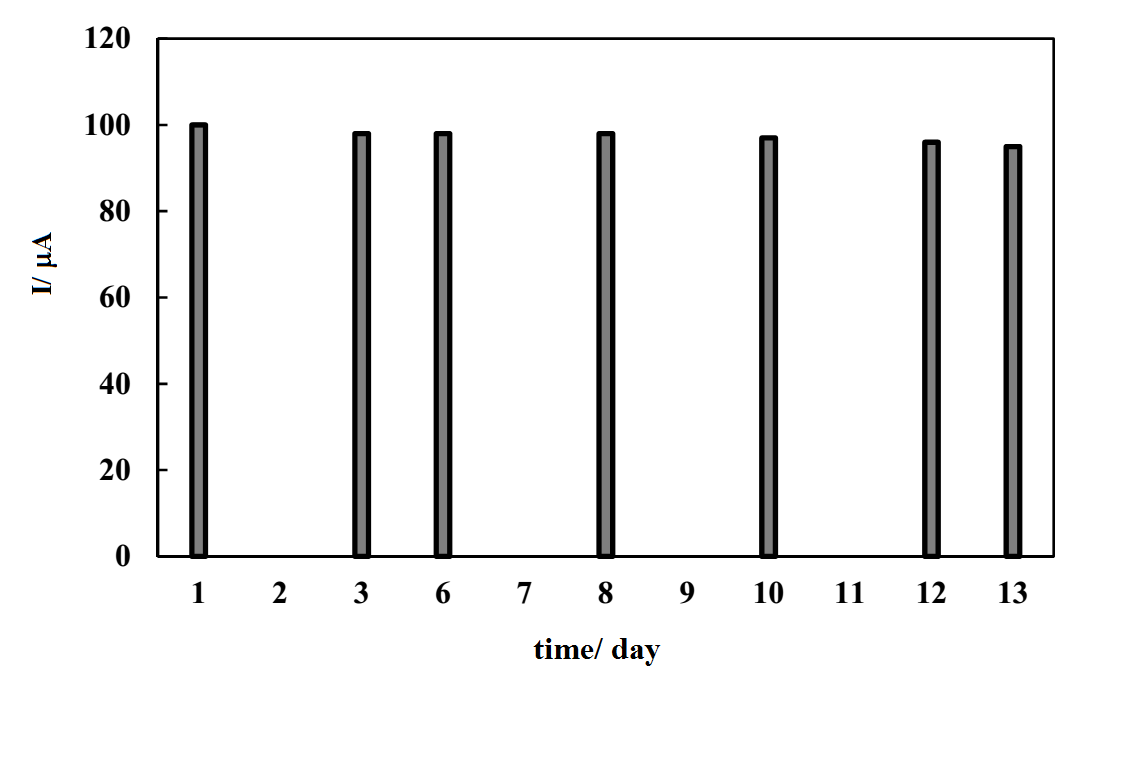


**Fig. S16.** Variation of DPV stripping signal of L-Arg-RGO/GCE in Pb(II) free KCl solution after storing at 4 °C for different time intervals. Accumulation conditions: KCl (pH 6) applying -0.8 V for 60 s in 3 nM Pb(II)
